# Effect of continuing versus stopping pre-stroke antihypertensive agents within 12 h on outcome after stroke: A subgroup analysis of the efficacy of nitric oxide in stroke (ENOS) trial

**DOI:** 10.1016/j.eclinm.2022.101274

**Published:** 2022-01-24

**Authors:** Lisa J. Woodhouse, Jason P. Appleton, Polly Scutt, Lisa Everton, Gwenllian Wilkinson, Valeria Caso, Anna Czlonkowska, John Gommans, Kailash Krishnan, Ann C. Laska, George Ntaios, Serefnur Ozturk, Stephen Phillips, Stuart Pocock, Kameshwar Prasad, Szabolcs Szatmari, Joanna M. Wardlaw, Nikola Sprigg, Philip M. Bath, ENOS Investigators

**Affiliations:** a Stroke Trials Unit, Mental Health & Clinical Neuroscience, Queen's Medical Centre, University of Nottingham, South Block D floor, Nottingham NG7 2UH UK; b Stroke, University Hospitals Birmingham NHS Foundation Trust, Edgbaston, Birmingham B15 2GW, UK; c Institute of Applied Health Research, College of Dental and Medical Sciences, University of Birmingham, Birmingham, UK; d Stroke, Nottingham University Hospitals NHS Trust, Nottingham NG7 2UH, UK; e Stroke Unit, Santa Maria della Misericordia Hospital, University of Perugia, Italy; f Institute of Psychiatry and Neurology, Warsaw, Poland; g Department of Medicine, Hawke's Bay District Health Board, Hastings, New Zealand; h Department of Clinical Science, Karolinska Institute, Danderyd Hospital, Stockholm, Sweden; i Department of Internal Medicine, School of Health Sciences, University of Thessaly, Greece; j Neurology, Selcuk University Faculty of Medicine, Konya, Turkey; k Division of Neurology, Department of Medicine, Dalhousie University, Halifax, Canada; l London School of Hygiene and Tropical Medicine, London, UK; m Rajendra Institute of Medical Sciences, Ranchi, Jharkhand 834009, India; n Department of Neurology, Clinical County Emergency Hospital, Targu Mures, Romania; o Centre for Clinical Brain Sciences, University of Edinburgh, Edinburgh, UK

## Abstract

**Background:**

It is not known whether to continue or temporarily stop existing antihypertensive drugs in patients with acute stroke.

**Methods:**

We performed a prospective subgroup analysis of patients enrolled into the Efficacy of Nitric Oxide in Stroke (ENOS) trial who were randomised to continue vs stop prior antihypertensive therapy within 12 h of stroke onset. The primary outcome was functional outcome, assessed with the modified Rankin Scale at 90 days by observers blinded to treatment assignment, and analysed with ordinal logistic regression.

**Findings:**

Of 4011 patients recruited into ENOS from 2001 to 2014, 2097 patients were randomised to continue vs stop prior antihypertensive treatment, and 384 (18.3%, continue 185, stop 199) were enrolled within 12 h of ictus: mean (SD) age 71.8 (11.8) years, female 193 (50.3%), ischaemic stroke 342 (89.1%) and total anterior circulation syndrome 114 (29.7%). As compared with stopping, continuing treatment within 12 h of onset lowered blood pressure by 15.5/9.6 mmHg (*p*<0.001/<0.001) by 7 days, shifted the modified Rankin Scale to a worse outcome by day 90, adjusted common odds ratio (OR) 1.46 (95% CI 1.01–2.11), and was associated with an increased death rate by day 90 (hazard ratio 2.17, 95% CI 1.24–3.79). Other outcomes (disability - Barthel Index, quality of life - EQ-visual analogue scale, cognition - telephone mini-mental state examination, and mood - Zung depression scale) were also worse with continuing treatment.

**Interpretation:**

In this pre-specified subgroup analysis of the large ENOS trial, continuing prior antihypertensive therapy within 12 h of stroke onset in a predominantly ischaemic stroke population was unsafe with worse functional outcome, disability, cognition, mood, quality of life and increased death. Future studies assessing continuing or stopping prior antihypertensives in the context of thrombectomy are awaited.

**Research in context**

**Evidence before this study**

We searched PubMed with the search terms “continue and stop”, “pre-stroke antihypertensive”, “prior antihypertensive”, “stroke”, and “clinical trial” for reports published before 18 May 2021. The only published results of clinical trials comparing continue with stopping prior antihypertensives in patients with stroke were those from the Continue or Stop Post-Stroke Antihypertensives Collaborative Study (COSSACS) and Efficacy of Nitric Oxide in Stroke (ENOS) trials. Meta-analysis of these two trials suggested that continuing prior antihypertensives was associated with a worse functional outcome as compared with stopping these temporarily in patients enrolled within 12 h of stroke onset.

**Added value of this study**

This subgroup analysis extends the meta-analysis findings; continuing pre-stroke antihypertensives in a population of mainly ischaemic stroke patients within 12 h of symptom onset was associated with more death, dependency, disability, cognitive impairment and mood disturbance and a worse quality of life at 90 days after stroke. When assessed in pre-specified subgroups, significant interactions were present for stroke severity and feeding status with a worse functional outcome present in those with more severe stroke and non-oral feeding at baseline who had been randomised to continue prior antihypertensive therapy.

**Implications of all the available evidence**

Continuing, rather than stopping, pre-stroke antihypertensives in ischaemic stroke patients presenting within 12 h of onset of stroke symptoms appears to be hazardous with worse clinical outcomes in multiple domains including functional outcome, disability, cognition, mood, quality of life and increased death.

Alt-text: Unlabelled box

## Introduction

Treating hypertension is effective at preventing first and recurrent stroke.^1^^,^^2^ As a result, many patients are taking blood pressure (BP)-lowering therapy at the time of any subsequent stroke. A common clinical problem is whether these drugs should be continued or stopped temporarily during the acute phase after stroke; a definitive answer remains unclear^3^ and clinical practice varies.^4^ Guidelines now recommend that patients should resume oral treatment once they are medically and neurologically stable, and can swallow safely.^5^

Two trials have examined this question. First, the multicentre Continue or Stop Post-Stroke Antihypertensives Collaborative Study (COSSACS) found that continuing antihypertensive drugs, as compared with stopping them, in 763 participants randomised within 48 h did not alter death or dependency at either two weeks or 6 months.^6^ Similarly, there was no difference in functional outcome in 2097 patients randomised within 48 h in the large Efficacy of Nitric Oxide in Stroke (ENOS) trial.^7^ However, there were non-significant tendencies to a worse outcome in those patients randomised to continue prior antihypertensive agents within two pre-defined time windows, i.e. within 6 h, and between 6 and 12 h, of stroke onset.^7^ When the two earlier time windows were pooled together, there was a positive interaction between time to randomisation (0–12 hrs, 12–24 hrs, 24–48 hrs), randomisation to continue or stop pre-stroke antihypertensives and functional outcome (p_interaction_=0.041). When examined in a meta-analysis of COSSACS and ENOS, worse functional outcome was seen in those randomised within 12 h (interaction between time to randomisation, randomised treatment and functional outcome *p* = 0.055).^8^ Here, we explore in more detail functional and other outcomes, and potential explanations, in ENOS participants randomised within 12 h of stroke onset.

## Methods

### Trial design

ENOS was an international multicentre parallel-group, randomised-controlled, patient-blinded, outcome assessor-blinded trial. Details of the design, statistical analysis plan, and main results for ENOS have been published previously.^7^^,^^9^, ^10^, ^11^ In brief, ENOS examined the safety and efficacy of glyceryl trinitrate (GTN) versus no GTN (single-blind delivery) in patients with acute ischaemic stroke or intracerebral haemorrhage. Patients who were taking antihypertensive therapy immediately prior to their stroke were also randomised to continue or stop this (open-label) in a partial factorial design. Both sets of interventions were given for 7 days. The primary outcome was the modified Rankin Scale (mRS) assessed by masked central telephone call at day 90. The trial recruited 4011 patients from 173 sites across 23 countries in 5 continents.^11^ The trial pre-specified that analyses would be assessed by time window: 0–6, 6–12, 12–24 and 24–48 h after stroke onset;(7) for the comparison of continue vs stop pre-stroke antihypertensive drugs (which involved approximately half the ENOS participants), we merged the first two (and smaller) time windows. Patients or relatives/carers provided written informed consent to participate. ENOS was registered (ISRCTN99414122) and approved by ethics committees or competent authorities in participating countries.

### Patients

Hospitalised patients with a clinical syndrome of a stroke due to ischaemic stroke or intracerebral haemorrhage were eligible for the trial if they were aged 18 or over, had a motor deficit in arm and/or leg, had a systolic blood pressure between 140 and 220 mmHg, and treatment could be started within 48 h of onset.^9^ The diagnosis of ischaemic or haemorrhagic stroke was confirmed with computed tomography (CT) or magnetic resonance imaging (MRI) done before, or soon after, enrolment.

Key exclusion criteria included: definite need to start, continue, or stop, BP-lowering medications; need for, or contraindication to, GTN; coma (Glasgow Coma Scale <8); pure sensory stroke; isolated dysphasia; preceding moderate or severe dependency (modified Rankin scale, mRS 3–5); confounding neurological or psychiatric disease; a condition mimicking stroke (e.g. hypoglycaemia, Todd's paresis); liver dysfunction (INR > 1.5, aminotransferase > 3 x normal) or renal dysfunction (creatinine > 3 x normal); severe concomitant medical condition; pregnancy or breast feeding; previous participation in ENOS; planned surgical intervention; or participation in another trial within 2 weeks of randomisation.

### Randomisation and masking

Written informed consent was obtained from each patient, or from a relative or independent physician if the patient lacked capacity, prior to enrolment and in accordance with national regulations. Investigators entered baseline and follow-up data into a database via a secure web-based randomisation system (www.enos.ac.uk). Data were checked to confirm the eligibility of the patient, and the system then assigned a patient to continue or stop BP drugs with the use of stratification (stroke pathological type, country, GTN vs no GTN), minimisation (age ≥70 years, male sex, history of hypertension, history of previous stroke, history of diabetes mellitus, stroke severity as Scandinavian Stroke Scale <30, total anterior circulation syndrome,^12^ systolic BP >160 mmHg, no treatment with alteplase, oral feeding not possible, and time to randomisation <24 h), and simple randomisation (in 5% of patients). The randomisation algorithm then presented a treatment allocation as either ‘Continue’ or ‘Stop’ prior antihypertensive drugs. This randomisation procedure was used to ensure participants were assigned evenly between treatment arms of the trial.

### Treatment

Treatment consisted of continuing or stopping antihypertensive drugs taken regularly prior to the stroke. In patients who were assigned to continue their BP drugs, these were prescribed open label via the site's hospital system using local pharmaceutical supplies; patients assigned to stop their BP drugs were not given any of these for the following 7 days. In patients who were dysphagic, drugs were administered, where possible, via a nasogastric tube.

Treatment was given in addition to standard care; thrombolysis was permitted in patients with ischaemic stroke according to local practice guidelines at the recruiting site. Study agents could be stopped if the patient withdrew consent, for safety reasons, or if unacceptable adverse events developed. Non-trial nitrates and other antihypertensive agents were avoided during the 7-day treatment period unless deemed necessary by the local investigator. Systematic use of antihypertensive agents (all patients, after 7 days), and oral antithrombotic and lipid lowering agents (patients with ischaemic stroke) were recommended for secondary prevention.

### Outcomes

The primary outcome was the seven level mRS,^7^ as recommended by the European Stroke Organisation,^13^ with assessment at day 90. The effect of treatment on mRS was assessed in pre-specified subgroups: age, sex, history of hypertension, previous stroke, atrial fibrillation, nitrate use, systolic BP, stroke type, stroke severity, stroke syndrome, presence of ipsilateral carotid stenosis, use of alteplase, and randomisation to GTN vs. no GTN.

BP was measured three times prior to randomisation and in the morning (twice) for the 7 days of treatment using a validated automated monitor (Omron 705CP).^14^ Between-visit BP variability was defined as the standard deviation of systolic BP over the 7 days of treatment. Secondary outcomes at day 7 included: intracerebral haemorrhage, recurrent stroke, deterioration,^10^ and impairment (Scandinavian Stroke Scale, SSS, and calculated National Institutes of Health Stroke Scale, NIHSS^15^). Resource utilisation was assessed on discharge from (or death in) hospital: length of stay; assessment/treatment by a physiotherapist, occupational therapist or speech therapist; and discharge destination (ordered categorical scale: died in hospital, still in hospital, in rehabilitation hospital, in nursing home, in residential home, at carer's home, or at home alone or with partner-carer).

Secondary outcomes at day 90 included: activities of daily living (Barthel Index), quality of life (health utility status, derived from EuroQol-5 dimensions-3 levels, EQ-5D; EQ-visual analogue scale, EQ-VAS), cognition (telephone mini-mental state examination, MMSE; modified telephone interview cognition scale, TICS-M; category fluency as animal naming), mood (Zung depression scale, short-form, ZDS^16^), recurrent stroke, and disposition (died, still in hospital, readmitted to hospital, in nursing home, in residential home, at carer's home, at home alone or with partner-carer).

Safety measures included all-cause death and serious adverse events, the latter coded in an ordered categorical scale^17^: fatal SAE, non-fatal SAE, no SAE; pneumonia or chest infection (derived from reported SAEs); and hypotension and hypertension by day 7.

### Statistics

The sample size calculation for the comparison of continuing vs stopping pre-stroke antihypertensives within the ENOS trial was 1750 to allow a shift in mRS with odds ratio (OR) 0.80 or 1.25 to be detected with 80% power.^10^ All ordinal or continuous measures include a separate score for death, as is standard for mRS and EQ-5D/Health utility status; death was coded as −5 for Barthel index; −1 for animal naming, EQ-VAS, MMSE and TICS; 0 for EQ-5D/Health utility status; 6 for mRS; and 102.5 for ZDS.^18^^,^^19^ Data are shown as number (%), median [interquartile range] or mean (standard deviation). Comparisons between groups used binary logistic regression, Cox proportional hazards regression, ordinal logistic regression (OLR) or multiple linear regression; results are given as OR or difference in means (DIM), with 95% confidence intervals (95% CI) and significance, with *p*<0.05 being considered significant. The assumption of proportionality of odds for OLR was tested using the likelihood ratio. Analyses are shown both unadjusted, and adjusted for age, sex, pre-morbid mRS, previous stroke, history of diabetes, prior use of nitrates, final diagnosis, SSS, total anterior circulation syndrome (TACS), SBP, use of thrombolysis, feeding status, time to randomisation, and randomisation to GTN vs. no GTN. Pre-specified subgroup analyses were performed by adding an interaction term to an unadjusted OLR model. In addition, to assess whether baseline imaging markers of small vessel disease and brain frailty^20^ influence the treatment effect of continuing vs. stopping pre-stroke antihypertensives, an interaction term was added to an unadjusted OLR model in post-hoc analyses. A pre-specified global outcome (comprising ordered categorical or continuous data for mRS, Barthel index, ZDS, TICS-M and Health utility status) was analysed using the Wei-Lachin test.^21^, ^22^, ^23^ To establish whether feeding status or pneumonia influenced the treatment effect of continue vs. stop on the primary outcome, we performed a causal mediation analysis.^24^ No adjustment was made for multiplicity of testing. Analyses were performed using SAS version 9.3/9.4.

### Role of the funding source

The funders had no role in study design, in the collection, analysis, and interpretation of data, in the writing of the report, and in the decision to submit the paper for publication. PMB, JPA and LJW had access to the data. PMB is corresponding author and took the decision to submit for publication.

## Results

### Patients

Of the 4011 participants randomised into ENOS, 2097 were randomised to either stop or continue prior antihypertensive treatment and 384 (18.3%; continue 185, stop temporarily 199) of these were recruited within 12 h of symptom onset (Table 1). Participants in this target time window of <12 h were taking a median of 2 [1, 2] antihypertensive medications. Mean (SD) age was 71.8 (11.8) years, 193 (50.3%) were female, 212 (55.2%) were recruited from the UK, and 342 (89.1%) had an ischaemic stroke. Mean baseline Scandinavian Stroke Scale was 32.7 (12.4), 114 (29.7%) had a total anterior circulation syndrome, 36.7% required non-oral feeding, and the mean baseline BP was 168.7 (19.9) / 89.8 (13.0) mmHg. The index event was ischaemic stroke in 342 (89.1%) participants, whilst 39 (10.2%) had an intracerebral haemorrhage. Of participants with an ischaemic stroke, 79 (23.1%) received intravenous thrombolysis. In those randomised within 12 h of symptom onset, there were no differences in baseline characteristics between continue and stop groups. As compared with those randomised between 12 and 48 h, participants randomised within 12 h of onset were more likely to be younger, have less pre-morbid dependency (mRS score >0), have a feeding status of ‘nothing’, have a higher diastolic BP and be randomised to GTN than no GTN (Table 1).

**Table 1:** Baseline characteristics in continue/stop patients randomised within 12 h by treatment group, and those randomised beyond 12 h. Data are number (%), median [interquartile range] or mean (standard deviation). Comparisons are between those randomised within and beyond 12 h.

	Continue	Stop	2p	>12 h	≤12 h	2p	
Number of patients	185	199		1713	384		
Age (years) †	72.8 (12.3)	70.9 (11.2)	0.11	73.1 (11.0)	71.8 (11.8)	0.037	
Sex, male (%) †	85 (45.9)	106 (53.3)	0.15	877 (51.2)	191 (49.7)	0.61	
**Geographical region**							
Asia	11 (5.9)	12 (6.0)	0.75	179 (10.4)	23 (6.0)	<0.0001	
Europe	46 (24.9)	50 (25.1)	–	311 (18.2)	96 (25.0)	–	
United Kingdom	106 (57.3)	106 (53.3)	–	1140 (66.5)	212 (55.2)	–	
Other	22 (11.9)	31 (15.6)	–	83 (4.8)	53 (13.8)	–	
mRS > 0†	56 (30.3)	50 (25.1)	0.26	578 (33.7)	106 (27.6)	0.020	
**Medical history (%)**							
Hypertension	178 (96.2)	193 (97.0)	0.68	1623 (94.7)	371 (96.6)	0.13	
Treated hypertension	185 (100.0)	198 (99.5)	0.33	1703 (99.4)	383 (99.7)	0.43	
Hyperlipidaemia	74 (40.0)	65 (32.7)	0.23	669 (39.1)	139 (36.2)	0.15	
Atrial fibrillation	48 (25.9)	51 (25.6)	0.94	467 (27.3)	99 (25.8)	0.55	
Diabetes†	36 (19.5)	49 (24.6)	0.22	399 (23.3)	85 (22.1)	0.63	
Previous stroke†	35 (18.9)	41 (20.6)	0.68	340 (19.8)	76 (19.8)	0.98	
TIA	36 (19.5)	34 (17.1)	0.81	282 (16.5)	70 (18.2)	0.20	
IHD	52 (28.1)	44 (22.1)	0.39	427 (24.9)	96 (25.0)	0.61	
PAD	6 (3.2)	10 (5.0)	0.51	61 (3.6)	16 (4.2)	0.36	
Smoking, current	30 (17.3)	36 (18.7)	0.74	297 (18.2)	66 (18.0)	0.95	
Alcohol >21 upw	7 (3.8)	10 (5.0)	0.20	87 (5.1)	17 (4.4)	0.64	
Nitrate therapy†	12 (6.5)	13 (6.5)	0.99	111 (6.5)	25 (6.5)	0.98	
**Treated high BP**^‡,∞^							
ACE-Inhibitor	92 (49.7)	82 (41.2)	0.17	825 (48.2)	174 (45.3)	0.59	
Angiotensin receptor antagonist	27 (14.6)	37 (18.6)	0.34	273 (15.9)	64 (16.7)	0.93	
Beta-receptor antagonist	71 (38.4)	80 (40.2)	0.71	669 (39.1)	151 (39.3)	0.92	
Calcium channel blocker	60 (32.4)	64 (32.2)	0.95	601 (35.1)	124 (32.3)	0.41	
Diuretic	61 (33.0)	69 (34.7)	0.73	605 (35.3)	130 (33.9)	0.61	
Alpha-receptor antagonist	9 (4.9)	12 (6.0)	0.62	125 (7.3)	21 (5.5)	0.31	
Centrally acting drug	2 (1.1)	3 (1.5)	0.71	27 (1.6)	5 (1.3)	0.53	
Other	4 (2.2)	3 (1.5)	0.52	16 (0.9)	7 (1.8)	0.080	
**No. of BP drugs**							
0	0	1 (0.5)	0.26	10 (0.6)	1 (0.3)	0.55	
1	87 (47.0)	84 (42.2)		744 (43.4)	171 (44.5)	–	
2	63 (34.1)	81 (40.7)		585 (34.2)	144 (37.5)	–	
3	25 (13.5)	28 (14.1)		282 (16.5)	53 (13.8)	–	
4	10 (5.4)	4 (2.0)		79 (4.6)	14 (3.6)	–	
5	0	1 (0.5)		12 (0.7)	1 (0.3)	–	
6	0	0		1 (0.1)	0	–	
Median [IQR]	2.0 [1.0, 2.0]	2.0 [1.0, 2.0]	0.77	2.0 [1.0, 2.0]	2.0 [1.0, 2.0]	0.34	
Mean (SD)	1.8 (0.9)	1.8 (0.8)	0.92	1.8 (0.9)	1.8 (0.8)	0.20	
**Fluids and feeding**							
Normal diet	66 (35.7)	73 (36.7)	0.83	675 (39.4)	139 (36.2)	<0.0001	
Soft diet	51 (27.6)	53 (26.6)		405 (23.6)	104 (27.1)	–	
Nasogastric tube	3 (1.6)	3 (1.5)		97 (5.7)	6 (1.6)	–	
Percutaneous feeding tube	1 (0.5)	0		6 (0.4)	1 (0.3)	–	
Intravenous/ subcutaneous fluids	31 (16.8)	28 (14.1)		367 (21.4)	59 (15.4)	–	
No feeding/fluids	33 (17.8)	42 (21.1)		163 (9.5)	75 (19.5)	–	
**Qualifying event (%)**†							
Ischaemic stroke	166 (89.7)	176 (88.4)	0.84	1490 (87.0)	342 (89.1)	0.70	
Haemorrhagic stroke	18 (9.7)	21 (10.6)	–	207 (12.1)	39 (10.2)	–	
Stroke type unknown	0	0	–	1 (0.1)	0	–	
Non-stroke	1 (0.5)	2 (1.0)	–	15 (0.9)	3 (0.8)	–	
Side of lesion, right (%)	94 (50.8)	94 (47.2)	0.44	886 (51.8)	188 (49.0)	0.40	
SSS (/58) †	31.8 (12.5)	33.5 (12.3)	0.19	32.9 (13.7)	32.7 (12.4)	0.79	
NIHSS (/42)^15^	12.0 (5.4)	11.3 (5.3)	0.19	11.5 (5.9)	11.6 (5.3)	0.79	
GCS <15 (%)	72 (38.9)	63 (31.7)	0.14	592 (34.6)	135 (35.2)	0.82	
**Clinical syndrome****^29^**							
TACS †	57 (30.8)	57 (28.6)	0.28	583 (34.0)	114 (29.7)	0.050	
PACS	72 (38.9)	78 (39.2)	–	552 (32.2)	150 (39.1)	–	
LACS	45 (24.3)	59 (29.6)	–	520 (30.4)	104 (27.1)	–	
POCS	11 (5.9)	5 (2.5)	–	58 (3.4)	16 (4.2)	–	
**IS aetiology**							
Cardioembolic	58 (34.9)	52 (29.5)	0.29	397 (26.6)	110 (32.2)	0.024	
Large vessel	33 (19.9)	44 (25.0)	0.26	340 (22.8)	77 (22.5)	0.93	
Small vessel disease	49 (29.5)	60 (34.1)	0.36	517 (34.7)	109 (31.9)	0.49	
Other	26 (15.7)	28 (15.9)	0.95	276 (18.5)	54 (15.8)	0.32	
**Haemodynamics**							
BP, Systolic (mmHg)†	167.8 (20.1)	169.6 (19.8)	0.37	166.7 (18.5)	168.7 (19.9)	0.059	
BP, Diastolic (mmHg)	89.0 (12.8)	90.5 (13.1)	0.28	88.0 (13.0)	89.8 (13.0)	0.014	
Heart rate (bpm)	77.5 (16.2)	78.2 (15.1)	0.67	77.0 (15.1)	77.9 (15.6)	0.28	
OTR (hours) †	7.3 (2.6)	7.3 (2.8)	0.97	29.7 (10.6)	7.3 (2.7)	<0.0001	
Thrombolysis (%)†	37 (20.0)	43 (21.6)	0.93	168 (9.8)	80 (20.8)	<0.0001	
**GTN Randomisation**							
GTN	96 (51.9)	113 (56.8)	0.34	825 (48.2)	209 (54.4)	0.026	
No GTN	89 (48.1)	86 (43.2)	–	888 (51.8)	175 (45.6)	–	
**Baseline scan (%)**							
Visible infarction	91 (49.2)	84 (42.2)	0.52	1000 (58.4)	175 (45.6)	<0.0001	
Visible haemorrhage	18 (9.8)	21 (10.6)	–	210 (12.3)	39 (10.2)	–	
No lesion seen	73 (39.9)	93 (46.7)	–	490 (28.7)	166 (43.5)	–	
Non-stroke lesion	1 (0.5)	1 (0.5)	–	6 (0.4)	2 (0.5)	–	

† Variable used in statistical adjustment 2p: 2-sided *p*-value; BP: blood pressure; bpm: beats per minute; GCS: Glasgow Coma Scale; GTN: glyceryl trinitrate; IHD: ischaemic heart disease; LACS: lacunar syndrome; mRS: modified Rankin Scale; NIHSS: National Institutes of Health Stroke Scale; OTR: time from onset to randomisation; PACS: partial anterior circulation syndrome; PAD: peripheral artery disease; POCS: posterior circulation syndrome; SSS: Scandinavian Stroke Scale; TIA: transient ischaemic attack; TACS: total anterior circulation syndrome; upw: units per week.

### Outcomes

As compared with stopping pre-stroke antihypertensives, continuing them significantly lowered BP by day 2 by 8.8/4.2 mmHg (*p*<0.001 / *p* = 0.003) and the difference increased throughout the treatment period to day 7: 15.5 / 9.6 mmHg (*p*<0.001 / *p*<0.001, Supplementary Figure 1). On-treatment between-visit BP variability adjusted for mean BP was higher in those randomised to continue vs. stop pre-stroke antihypertensives: DIM 1.50, 95% CI 0.11 to 2.90, *p* = 0.035.

Although there were no differences in symptomatic intracranial haemorrhage or recurrent stroke rates by day 7, significantly more patients randomised to continue vs. stop prior antihypertensives had deteriorated neurologically: 27 (14.7%) vs. 15 (7.5%), OR 2.37 (95% CI 1.16 to 4.87; *p* = 0.018; Table 2). More patients randomised to continue prior antihypertensives died in hospital or were discharged to an institution than those who stopped such medication: 76 (41.8%) vs. 53 (26.8%), OR 2.29, 95% CI 1.37 to 3.84, *p* = 0.002. There was no difference in speech and language involvement or length of stay between treatment groups.

**Table 2:** Primary and secondary outcomes, and safety measures, at days 7 and 90 in patients randomised within 12 h of stroke onset. Data are number (%), median [interquartile range] or mean (standard deviation). Comparisons between continue vs stop prior antihypertensives use adjusted binary logistic regression, ordinal logistic regression or multiple regression; results are odds ratio or mean difference, with 95% confidence intervals and significance.

	All	Continue	Stop	OR/DIM (95%CI)	2p
*Modified Rankin Scale*					
Median (/6), primary outcome	3 [2, 4]	3 [2, 5]	3 [1, 4]	1.46 (1.01, 2.11)	0.044
mRS >2, adjusted	223 (58.5)	112 (61.2)	111 (56.1)	1.09 (0.68, 1.77)	0.71
*Day 7*					
sICH (%)	8 (2.1)	5 (2.7)	3 (1.5)	2.03 (0.33, 12.36)	0.44
Recurrent stroke (%)	14 (3.7)	9 (4.9)	5 (2.5)	2.11 (0.63, 7.12)	0.23
Deterioration (%)^10^	42 (11.0)	27 (14.7)	15 (7.5)	2.37 (1.16, 4.87)	0.018
SSS (/58)	39.2 (16.6)	37.4 (18.1)	40.9 (14.9)	−2.1 (−4.4, 0.3)	0.083
NIHSS (/42)^15^	8.4 (6.7)	8.9 (7.1)	8 (6.3)	0.3 (−0.6, 1.3)	0.47
*Hospital and discharge*					
Length of stay (days)	9 [7,18]	10 [7,18]	9 [6,17]	1.3 (−2.1, 4.7)	0.46
Death or institution (%)	129 (34.0)	76 (41.8)	53 (26.8)	2.29 (1.37, 3.84)	0.0016
Speech therapy (%)	171 (45.0)	82 (45.1)	89 (44.9)	0.97 (0.60, 1.56)	0.91
*Day 90*					
Barthel Index (/100)	65.4 (39.1)	59.0 (43.0)	71.3 (34.2)	−9.0 (−15.2, −2.7)	0.0049
EQ-5D/HUS (/1)	0.5 (0.4)	0.4 (0.4)	0.5 (0.4)	−0.1 (−0.1, 0.0)	0.096
EQ-VAS (/100) (*N* = 341)	55.1 (32.4)	49.1 (35.3)	60.4 (28.7)	−8.4 (−14.2, −2.5)	0.0050
MMSE (*N* = 220)	10.8 (7.7)	9.6 (8.4)	12 (6.7)	−1.9 (−3.4, −0.3)	0.021
TICS-M (*N* = 218)	14.2 (10.8)	12.7 (11.6)	15.8 (9.8)	−2.3 (−4.6, −0.1)	0.038
Verbal Fluency (/∞) (*N* = 245)	8.8 (7.3)	7.9 (7.7)	9.7 (7.0)	−1.1 (−2.6, 0.4)	0.16
ZDS (/100) (*N* = 315)	60.1 (25.4)	64.8 (26.7)	55.8 (23.4)	7.9 (3.2, 12.7)	0.0011
Death or institution (%)	105 (27.6)	62 (34.1)	43 (21.7)	1.94 (1.14, 3.30)	0.014
*Safety*					
Patients with SAE (%)					
Day 7	54 (14.1)	28 (15.1)	26 (13.1)	1.18 (0.64, 2.19)	0.59
Day 90	108 (28.1)	60 (32.4)	48 (24.1)	1.44 (0.89, 2.34)	0.14
Died (%)					
By day 7	9 (2.3)	8 (4.3)	1 (0.5)	11.17 (1.2, 104.22)	0.034
In hospital	36 (9.5)	25 (13.7)	11 (5.6)	2.78 (1.2, 6.44)	0.017
By day 90	59 (15.5)	40 (21.9)	19 (9.6)	2.77 (1.41, 5.46)	0.0032
Day 7 (%)					
Headache	41 (10.7)	19 (10.3)	22 (11.1)	1.02 (0.51, 2.04)	0.95
Hypotension	11 (2.9)	7 (3.8)	4 (2.0)	3.46 (0.76, 15.73)	0.11
Hypertension	32 (8.4)	13 (7.1)	19 (9.5)	0.88 (0.4, 1.93)	0.75

2p: 2-sided p-value; EQ-5D/HUS: European quality of life 5 dimensions / health utility score; EQ-VAS: European quality of life visual analogue scale; mRS: modified Rankin Scale; NIHSS: National Institutes of Health Stroke Scale; sICH: symptomatic intracranial haemorrhage; SSS: Scandinavian Stroke Scale; ZDS: Zung depression scale.

**Figure 1:**
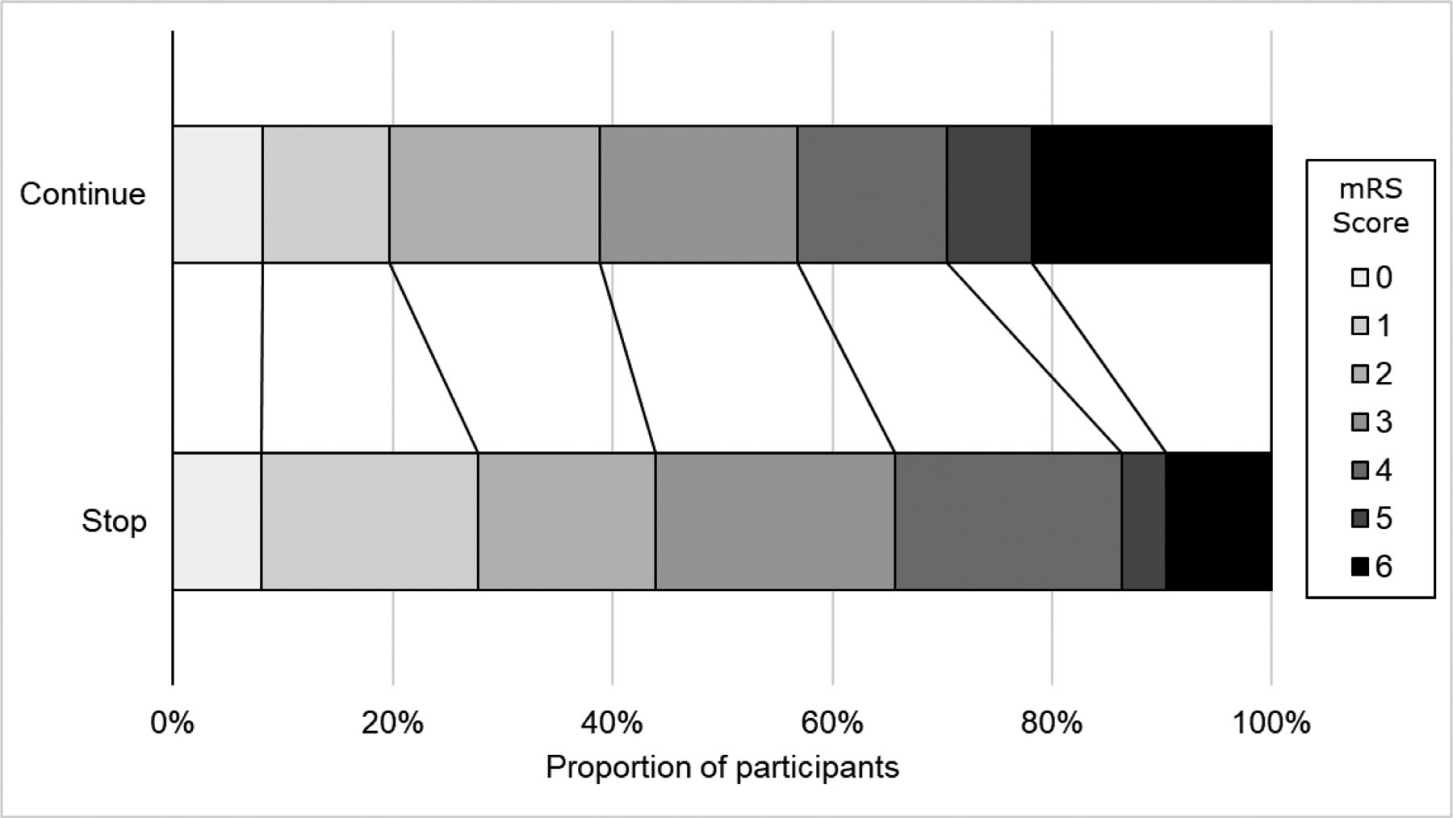
Comparison in distribution of seven-level modified Rankin Scale between continue versus stop prior antihypertensives at day 90. Continuing prior antihypertensives was associated with a worse functional outcome, adjusted common odds ratio 1.46 (95% CI 1.01–2.11, *p* = 0.044), unadjusted common odds ratio 1.56 (95% CI 1.09–2.22, *p* = 0.015).

**Figure 2:**
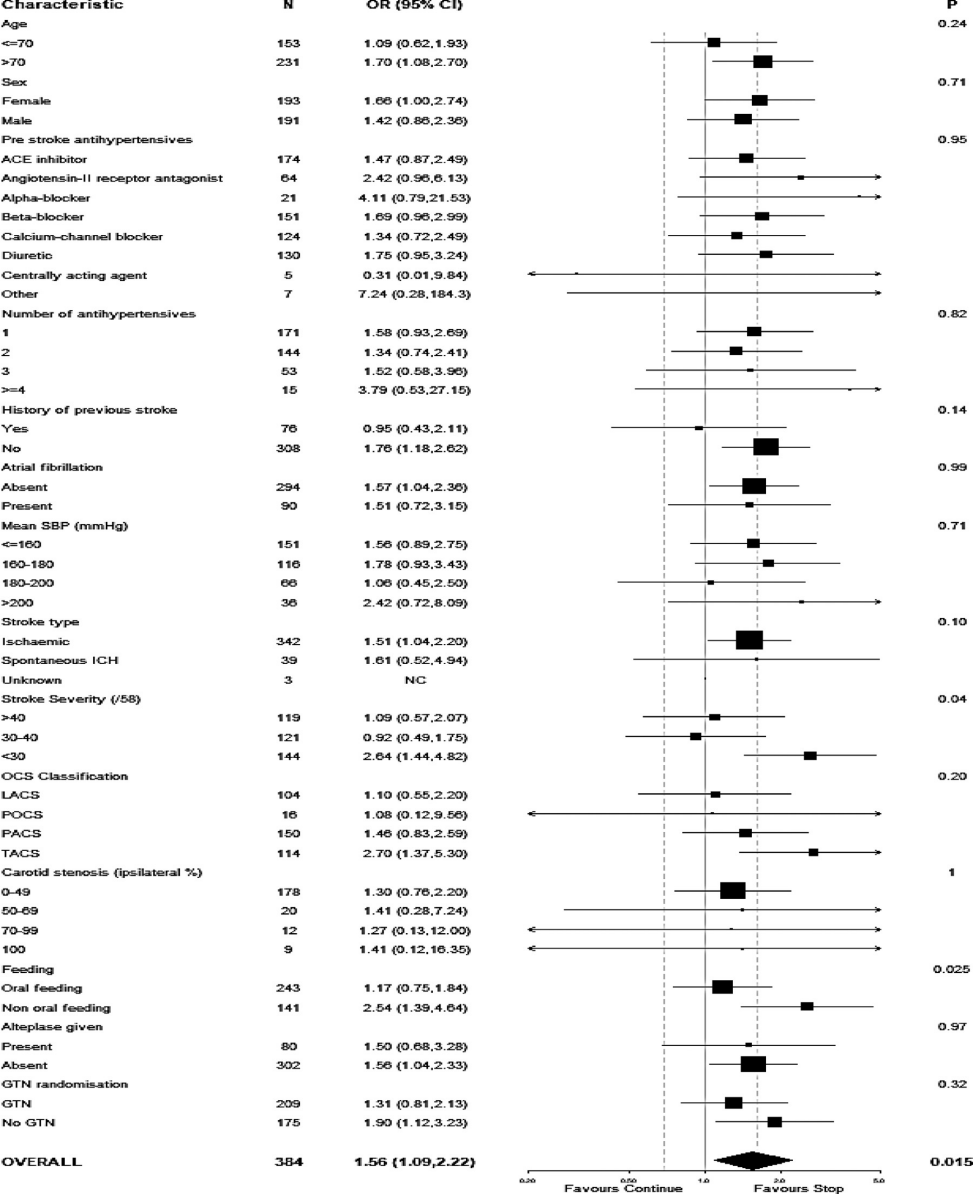
Subgroup analysis of effects on functional outcome at 90 days for continue versus stop prior antihypertensives for patients enrolled within 12 h of stroke onset. Ordinal logistic regression was used to produce odds ratios with 95% confidence intervals for each subgroup. Two-sided *p* values are for the interaction between subgroup and allocated treatment. OCSP: Oxfordshire Community Stroke Project.^29^ Significant interactions were present for stroke severity (severe vs moderate/mild) and feeding (non-oral vs oral).

The primary outcome of death and dependency measured using the mRS at day 90 revealed an unfavourable shift to poor outcome in those randomised to continue vs. stop prior antihypertensives in both adjusted and unadjusted analyses: adjusted OR 1.46, 95% CI 1.01 to 2.11, *p* = 0.044; unadjusted OR 1.56, 95% CI 1.09 to 2.22, *p* = 0.015 (Table 2, Figure 1). When the treatment effect on outcome was assessed in pre-specified subgroups, significant interactions were noted for stroke severity and feeding status (Figure 2). Those with more severe strokes (SSS<30) who were randomised to continue their prior antihypertensives had an unfavourable shift to more death and dependency (p_interaction_=0.040). Likewise, those with non-oral feeding at randomisation who were randomised to continue their antihypertensives had a shift to worse functional outcome (p_interaction_=0.025). No other significant interactions were noted, in particular there was no interaction for GTN vs no GTN, or across different classes of pre-stroke antihypertensives (Figure 2).

Post-hoc analyses of baseline imaging features found no interaction between small vessel disease or brain frailty scores and continue vs. stop pre-stroke antihypertensives on the primary outcome: small vessel disease score p_interaction_=0.63; brain frailty score p_interaction_=0.95.

Causal mediation analysis revealed that baseline non-oral feeding status was associated with increased death and dependency at 90 days, and with increased rates of pneumonia. In turn, pneumonia was associated with increased death and dependency at 90 days. Although continuing pre-stroke antihypertensives was associated with increased death and dependency at 90 days, it did not influence pneumonia rates and there was no interaction between treatment and pneumonia on functional outcome (Supplementary Figure 2).

As compared with stopping prior antihypertensives, continuing them was associated with worse disability (Barthel Index), quality of life (EQ-VAS), cognitive impairment (MMSE and TICS-M) and mood disturbance (ZDS), and more death or institutionalisation, at day 90 (Table 2). In a global analysis comprising mRS, Barthel Index, Health Utility Score, TICS-M and ZDS, continuing pre-stroke antihypertensives was associated with a tendency towards worse clinical outcome that just missed statistical significance (Mann-Whitney difference 0.05, 95% CI 0.00–0.09, *p* = 0.057, Supplementary Figure 3). A global analysis of cognitive outcomes demonstrated that continuing pre-stroke antihypertensives was associated with worse global cognitive outcome (Mann-Whitney difference 0.08, 95% CI 0.01–0.14, *p* = 0.02, Supplementary Figure 4).

**Figure 3:**
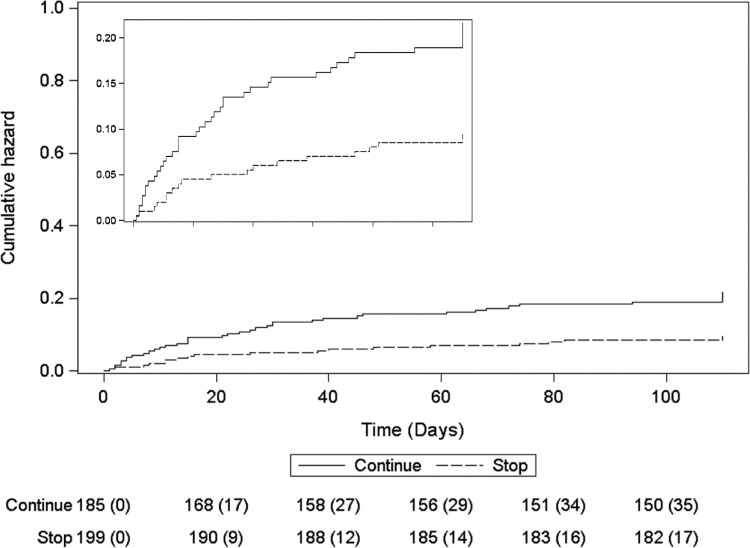
Survival curves over the 90 days of follow-up: continue versus stop prior antihypertensives. Continuing prior antihypertensives was associated with increased death: hazard ratio 2.17 (95% confidence interval 1.24–3.79; *p* = 0.007).

Participants who continued pre-stroke antihypertensive therapy had more fatal SAEs at day 90 and a non-significant tendency to more severe SAEs (using an ordered categorical scale) than those randomised to stop such medication (Supplementary Table 1 and Supplementary Figure 5). Although the rate of pneumonia did not differ between the treatment groups (Supplementary Table 2), there was a tendency towards an increase in fatal pneumonia if antihypertensives were continued, but this did not meet statistical significance (8/185 vs 2/199, *p* = 0.054). Continuing pre-stroke antihypertensives was significantly associated with increased death at day 7, in hospital, and at day 90 as compared with stopping such therapy (Table 2, Figure 3). Rates of headache, hypotension and hypertension by day 7 did not differ between treatment groups.

## Discussion

In this ENOS subgroup analysis we have demonstrated that continuing pre-stroke antihypertensives in a predominantly ischaemic stroke population within 12 h of symptom onset was associated with worse clinical outcomes across multiple domains and in global analyses up to 90 days after stroke including death, death or dependency, disability, cognition, quality of life and mood. Significant interactions for mRS were seen between continuing/stopping antihypertensives and stroke severity and non-oral feeding. Continuing pre-stroke antihypertensives was associated with more fatal SAEs and a non-significant tendency to more severe SAEs and non-fatal pneumonia.

These findings build upon the results of a meta-analysis of two trials demonstrating that continuing pre-stroke antihypertensives within 12 h of stroke onset may be hazardous.^8^ Although ENOS and COSSACS were neutral, both individually and when assessed in an individual patient data meta-analysis, the meta-analysis revealed that those randomised to continue antihypertensives within 12 h of stroke onset had an associated shift to unfavourable functional outcome at 90 and 180 days. Here, we have demonstrated that this negative effect is worse in patients with more severe stroke and is also seen across multiple domains manifest as worse disability, cognition, quality of life and mood at day 90, and increased death in hospital, at 7 and 90 days.

Several mechanisms can be postulated as to why continuing pre-stroke antihypertensives may be hazardous early after stroke. First, continuing pre-stroke antihypertensives in those with non-oral feeding was associated with worse functional outcome, which is a surrogate for stroke severity. Further, the positive interaction between treatment effect, outcome and stroke severity suggests that in severe stroke patients, continuing pre-stroke antihypertensives is hazardous. Second, given that the majority of participants had suffered an ischaemic stroke as their index event and 30% of participants had a TACS, a large proportion of this severe ischaemic stroke population will have had a large vessel occlusion. ENOS was conducted between 2001 and 2014 at a time when screening for large vessel occlusion and mechanical thrombectomy were not part of routine clinical practice. Therefore, the population presented here may represent those patients with non-recanalised large vessel occlusion. Lowering BP in the context of a blocked large intracranial artery may reduce cerebral blood flow distal to the occlusion by compromising the collateral circulation and extending the ischaemic core. A small trial (*n* = 163) suggested that inducing hypertension (systolic BP 200 mmHg) using intravenous phenylephrine in patients ineligible for revascularisation therapy within 24 h of onset improved neurological status by day 7 and was associated with less death and dependency at 90 days.^25^ Phenylephrine resulted in collateral enhancement at day 7 on MRI perfusion, supporting the rationale for not acutely lowering BP in the context of untreated large vessel occlusion. Further, in the enhanced control of hypertension and thrombolysis stroke study (ENCHANTED) BP arm, intensive lowering of BP in patients undergoing thrombolysis for acute ischaemic stroke was associated with worse clinical outcomes in those with large vessel occlusion on baseline imaging.^26^

Early continuation of anti-hypertensives was associated with worse clinical outcomes, whilst in patients randomised 12–48 h after ictus a neutral effect was observed in the aforementioned meta-analysis.^8^ In the crucial first hours after stroke, absolute BP changes and increased BP variability may have a greater influence on the ischaemic penumbra than on established infarction^27^ and on unstable haemorrhage than stable clot,^28^ which may account for the time-dependent effect on clinical outcome observed.

This secondary analysis of the ENOS trial has several strengths. First, the data come from a high-fidelity randomised controlled trial with international recruitment, wide inclusion criteria, delivery across a variety of stroke services and near-complete follow-up of the primary outcome. Second, deep phenotyping of participants and collection of intermediate outcomes allowed potential mechanisms to be examined. And last, collection of multiple outcome domains at final follow-up allowed the effect of treatment to be assessed on a global outcome.

There are important caveats. First, the results are based upon only 384 patients and analyses are, therefore, potentially underpowered and may represent a false negative (vs neutral) result. However, the internally consistent results suggest the results are real. Second, this is a subgroup analysis; positive or negative subgroups are not uncommon in large trials and have been shown, in several instances, to be ultimately due to chance. Third, although the inclusion criteria were wide, some patients will have been excluded, especially those with very high BP or very severe stroke. Hence the results cannot be extrapolated to all patients with strokes. Fourth, a proportion of patients may have been prescribed antihypertensives prior to their enrolment in ENOS but were not taking them due to non-compliance. In such participants then randomised to continue their pre-stroke antihypertensives, this would be akin to restarting or starting these medications rather than continuing them. Fifth, we did not investigate clustering in the data by site, country and continent. The majority of patients recruited were from the UK and therefore the findings are most generalisable to UK clinical practice. Last, the trial recruited from 2001 to 2014, spanning a rapid expansion and improvement in stroke care. As such, imaging data on large vessel occlusion and recanalisation status, and interventions such as thrombectomy were not routinely performed.

In summary, this pre-specified ENOS secondary analysis has demonstrated that continuing pre-stroke antihypertensives in patients with predominantly ischaemic stroke within 12 h of symptom onset was associated with worse functional outcome, disability, cognition, quality of life, mood and increased death. Further, continuing pre-stroke antihypertensives might have been particularly hazardous in those patients with a severe stroke and those with non-oral feeding at presentation. Future research assessing continuing or stopping pre-stroke antihypertensives in those patients with large vessel occlusion treated with mechanical thrombectomy stratified by reperfusion status may prove illuminating. Although there remains uncertainty and whilst awaiting further research,^29^ clinicians may wish to consider whether pre-stroke antihypertensives should be withheld for the first 12 h and until a secure feeding route has been established.

### Data sharing statement

The data referred to in this manuscript are available from the corresponding author upon reasonable request.

### Authors’ contributions

LJW, JPA and PMB had access to the data. LJW and JPA performed statistical analyses. JPA wrote the first draft. All authors commented upon and edited the manuscript. PMB is corresponding author and took the decision to submit for publication.

### Funding

ENOS was funded by the UK Medical Research Council (G0501797), and Bupa, and supported by the Stroke Association.

## Declaration of interests

JPA was supported in part by National Institute of Health Research (NIHR) TARDIS (10/104/24) and British Heart Foundation RIGHT-2 (CS/14/4/30972) and is supported by a NIHR Health and Care Research Scholarship. JMW was supported, in part, by the Scottish Funding Council through the SINAPSE Collaboration and the UK Dementia Research Institute which receives funding from DRI Ltd, funded by the UK Medical Research Council, Alzheimer's Society and Alzheimer's Research UK. PMB is Stroke Association Professor of Stroke Medicine and a NIHR Senior Investigator. All other authors report no declarations.

## Appendix Supplementary materials

Image, application 1
