# Supplementary material for: Effect of continuing versus stopping pre-stroke antihypertensive agents within 12 h on outcome after stroke: A subgroup analysis of the efficacy of nitric oxide in stroke (ENOS) trial
Source: eClinicalMedicine. 2022 Jan 24;44:101274. doi: 10.1016/j.eclinm.2022.101274 (PMC8790472; doi:10.1016/j.eclinm.2022.101274)
Supplement: Supplementary file 1 [file mmc1.docx]

**Supplementary Material for: Effect of continuing versus stopping pre-stroke antihypertensives within 12 hours on outcome after stroke: a subgroup analysis of ENOS**

1. Efficacy of Nitric Oxide in Stroke (ENOS) trial steering and international advisory committees
2. Supplementary tables
3. Supplementary figures
4. Statistical analysis plan
5. **Efficacy of Nitric Oxide in Stroke (ENOS) trial steering and international advisory committees**

**Trial Steering Committee:**

D Thomas (Independent Chair to 2006, UK), G Venables (Independent Chair from 2006, UK), P Amarenco (Independent Physician, France), K Muir (Independent Physician, UK), P M W Bath (Chief Investigator, UK), N Sprigg (Deputy Chief Investigator, UK), E Berge (Norway), K R Lees (UK), S Pocock (Statistician from 2003, UK), A Shone (Sponsor’s Representative, UK), A Skene (Statistician to 2003, UK), J M Wardlaw (Neuroradiologist, UK), D Whynes (Health economist, UK).

**International Advisory Committee:**

P M W Bath (Chair, UK), E Berge (Norway), M Beridze (Georgia), C Bladin (Australia), V Caso (Italy), C Chen/H M Chang (Singapore), H Christensen (Denmark), R Collins (Eire), A Czlonkowska (Poland), E Díez-Tejedor (Spain), A El Etribi (Egypt), A R Ghani (Malaysia), J Gommans (New Zealand), A C Laska (Sweden), K R Lees (UK), J Navarro (Philippines), G Ntaios (Greece), S Ozturk (Turkey), S Phillips (Canada), K Prasad (India), H A de Silva (Sri Lanka), S Szatmari (Romania), L Wong (Hong Kong), Y-J Wang (China).

**2. Supplementary tables**

**Supplementary Table 1**. Number of patients with serious adverse events during follow-up to day 90: continue versus stop pre-stroke antihypertensive drugs in patients enrolled within 12 hours of stroke onset.

| Cause | Any |  |  | Fatal |  |  |
| --- | --- | --- | --- | --- | --- | --- |
|  | Continue | Stop | P | Continue | Stop | P |
| Complication of initial stroke | 9 (4.9) | 2 (1.0) | 0.031 | 8 (4.3) | 2 (1.0) | 0.054 |
| Extension of initial stroke | 8 (4.3) | 4 (2.0) | 0.25 | 5 (2.7) | 0 | NC |
| Symptomatic intracranial haemorrhage | 5 (2.7) | 5 (2.5) | 1.00 | 1 (0.5) | 2 (1.0) | 1.00 |
| Recurrent stroke | 2 (1.1) | 8 (4.0) | 0.11 | 1 (0.5) | 1 (0.5) | 1.00 |
| Myocardial infarction | 3 (1.6) | 2 (1.0) | 0.68 | 2 (1.1) | 0 | NC |
| Sudden cardiac death | 0 | 1 (0.5) | NC | 0 | 1 (0.5) | NC |
| Atrial fibrillation | 1 (0.5) | 4 (2.0) | 0.37 | 0 | 1 (0.5) | NC |
| Carotid endarterectomy | 2 (1.1) | 1 (0.5) | 0.61 | 0 | 0 | NC |
| Other cardiovascular event | 9 (4.9) | 11 (5.5) | 0.77 | 3 (1.6) | 1 (0.5) | 0.36 |
| Pulmonary embolism | 3 (1.6) | 0 | NC | 2 (1.1) | 0 | NC |
| Pneumonia | 9 (4.9) | 6 (3.0) | 0.35 | 8 (4.3) | 2 (1.0) | 0.054 |
| Other event | 22 (11.9) | 16 (8.0) | 0.21 | 8 (4.3) | 6 (3.0) | 0.49 |
| Total | 60 (32.4) | 48 (24.1) | 0.070 | 38 (20.5) | 16 (8.0) | 0.000 |

Data are number of patients (%). Comparison by Chi-square/Fisher’s exact test. NC: Not calculable. Definitions for some events are given above, and in the Statistical Analysis Plan.(27)

**Supplementary Table 2**. Number of patients with pneumonia and therapy input in hospital by oral vs. non-oral feeding in those continuing vs. stopping pre-stroke antihypertensives within 12 hours of stroke onset. Data are number of patients (%). Comparison by Chi-square/Fisher’s exact test.

|  |  |  | Continue |  |  |  | Stop |  | Total |
| --- | --- | --- | --- | --- | --- | --- | --- | --- | --- |
| Feeding (28) | Total | Oral | Non-oral | 2p | Total | Oral | Non-oral | 2p | 2p |
| Patients | 185 | 117 | 68 | - | 199 | 126 | 73 | - | - |
| Pneumonia | 9 (4.9) | 4 (3.4) | 5 (7.4) | 0.29 | 6 (3.0) | 1 (0.8) | 5 (6.8) | 0.026 | 0.35 |
| Fatal pneumonia | 8 (4.3) | 3 (2.6) | 5 (7.4) | 0.15 | 2 (1.0) | 0 (0.0) | 2 (2.7) | NC | 0.054 |
| Physiotherapy | 142 (78.0) | 81 (69.2) | 61 (93.8) | 0.00012 | 154 (77.8) | 84 (66.7) | 70 (97.2) | <0.0001 | 0.95 |
| Occupational therapy | 101 (55.5) | 53 (45.3) | 48 (73.8) | 0.00020 | 113 (57.1) | 51 (40.5) | 62 (86.1) | <0.0001 | 0.76 |
| Speech therapy | 82 (45.1) | 39 (33.3) | 43 (66.2) | <0.0001 | 89 (44.9) | 36 (28.6) | 53 (73.6) | <0.0001 | 0.98 |

NC: Not calculable.

**3. Supplementary Figures**

**Supplementary Figure 1**. Systolic and diastolic blood pressure over 7 days: continue versus stop pre-stroke antihypertensive drugs patients enrolled within 12 hours of stroke onset.

**
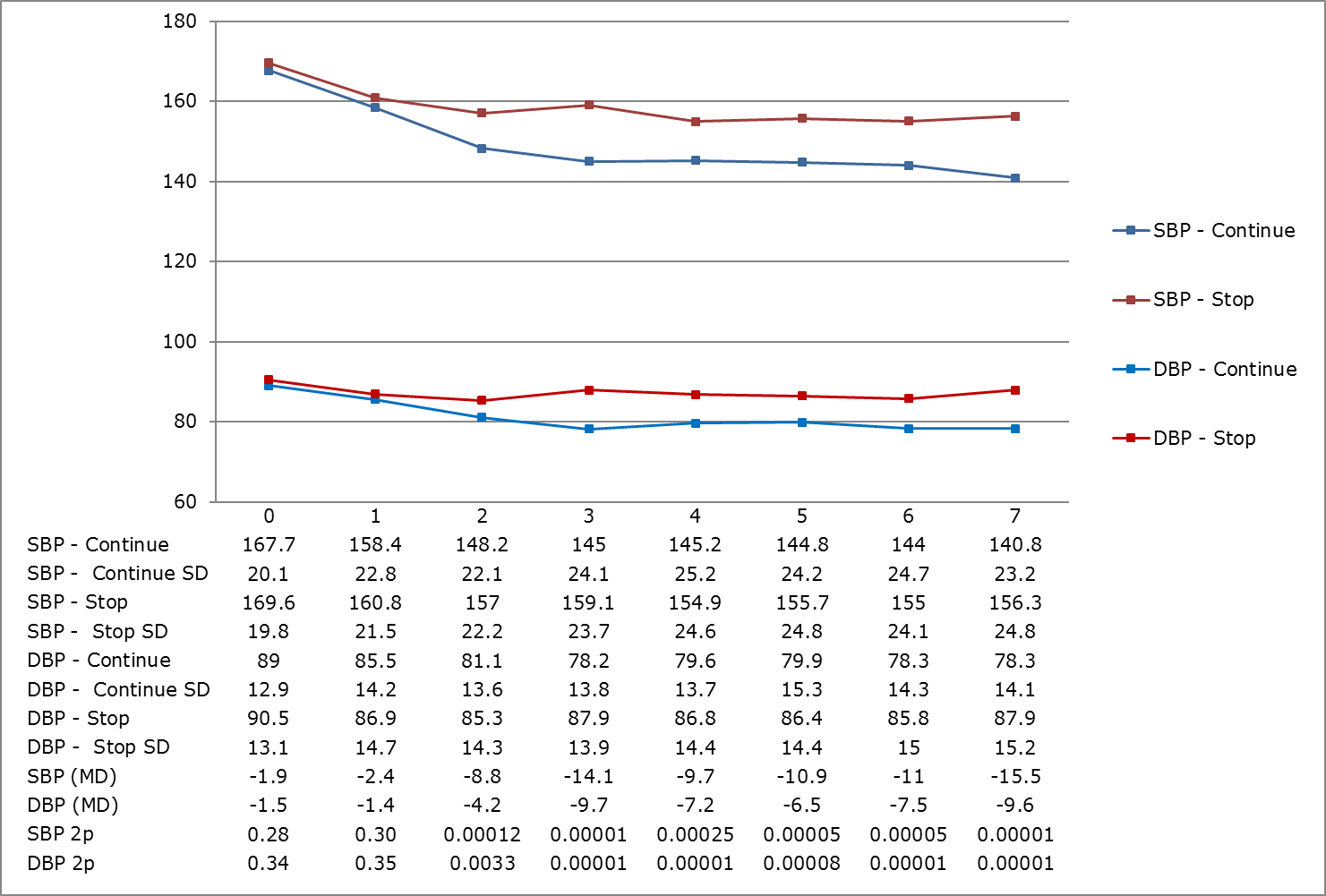
**

Day 0 is at randomisation; day 1 is 2 hours post-treatment. SBP DIM and DBP DIM signify mean difference in systolic and diastolic blood pressure between the two treatment groups. Comparisons by independent *t* test at each time point, and repeated measures analysis of variance: P=0.0002/<0.0001. Both systolic and diastolic blood pressure had diverged by day 2.

**Supplementary Figure 2**. Causal mediation analysis to assess the direct and indirect effects of non-oral feeding, pneumonia and continue vs. stop pre-stroke antihypertensives on functional outcome at day 90.

**Supplementary Figure 3**. Forest plot of global functional outcome comprising modified Rankin Scale, Barthel Index, Euro-QoL-5-dimension-3-level, modified telephone interview cognition scale and Zung depression scale. Analysis using Wei-Lachin test:(20) Mann-Whitney difference 0.05 (95% confidence intervals 0.00-0.09; p=0.057).


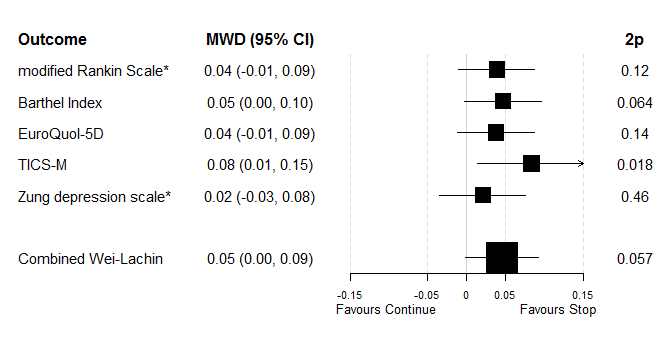


*scale inverted in line with other outcomes

**Supplementary Figure 4**. Forest plot of global cognitive outcome comprising modified telephone interview cognition scale, telephone mini-mental state examination and verbal fluency. Analysis using Wei-Lachin test:(20) Mann-Whitney difference 0.08 (95% confidence intervals 0.01-0.14; p=0.020).


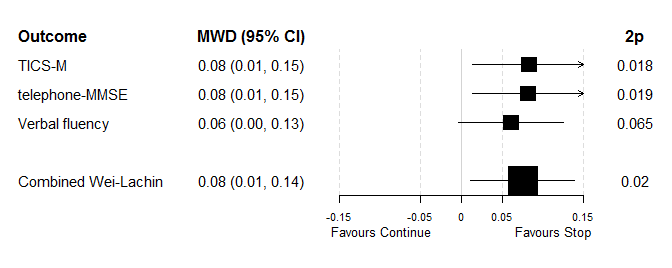


**Supplementary Figure 5**. Shift figure of SAEs (fatal, non-fatal SAE, no SAE) by continue vs stop prior antihypertensive agents. Comparison by ordinal logistic regression: Odds ratio 1.53 (95% confidence intervals 0.94-2.48; p=0.084).


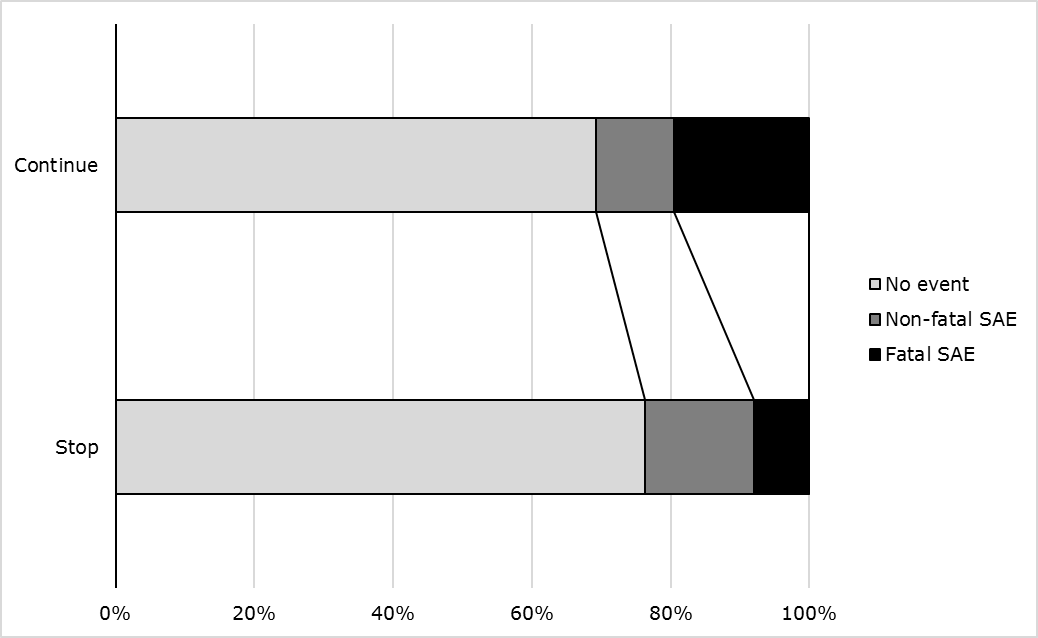


**4. Statistical analysis plan**

The statistical analysis plan for the ENOS trial is published. Pre-specified tables and figures relating to this manuscript are outlined below:

**Table 1**. Baseline characteristics in patients randomised within 12 hours by treatment group, and those randomised beyond 12 hours. Data are number (%), median [interquartile range] or mean (standard deviation). Comparisons are between those randomised within and beyond 12 hours.

|  | All >12 hours | All <12 hours | Continue | Stop | 2p |
| --- | --- | --- | --- | --- | --- |
| Number of patients |  |  |  |  |  |
| Age (years) † |  |  |  |  |  |
| Sex, male (%) † |  |  |  |  |  |
| Geographical region |  |  |  |  |  |
| Asia |  |  |  |  |  |
| Europe |  |  |  |  |  |
| United Kingdom |  |  |  |  |  |
| Other |  |  |  |  |  |
| mRS > 0 † |  |  |  |  |  |
| Medical history (%) |  |  |  |  |  |
| Hypertension |  |  |  |  |  |
| Treated hypertension |  |  |  |  |  |
| Hyperlipidaemia |  |  |  |  |  |
| Atrial fibrillation |  |  |  |  |  |
| Diabetes † |  |  |  |  |  |
| Previous stroke † |  |  |  |  |  |
| TIA |  |  |  |  |  |
| IHD |  |  |  |  |  |
| PAD |  |  |  |  |  |
| Smoking, current |  |  |  |  |  |
| Alcohol >21 upw |  |  |  |  |  |
| Nitrate therapy † |  |  |  |  |  |
| Treated high BP ‡∞ |  |  |  |  |  |
| ACE-Inhibitor |  |  |  |  |  |
| Angiotensin receptor antagonist |  |  |  |  |  |
| Beta-receptor antagonist |  |  |  |  |  |
| Calcium channel blocker |  |  |  |  |  |
| Diuretic |  |  |  |  |  |
| Alpha-receptor antagonist |  |  |  |  |  |
| Centrally acting drug |  |  |  |  |  |
| Other |  |  |  |  |  |
| No. of BP drugs |  |  |  |  |  |
| 0 |  |  |  |  |  |
| 1 |  |  |  |  |  |
| 2 |  |  |  |  |  |
| 3 |  |  |  |  |  |
| 4 |  |  |  |  |  |
| 5 |  |  |  |  |  |
| 6 |  |  |  |  |  |
| Median [IQR] |  |  |  |  |  |
| Mean (SD) |  |  |  |  |  |
| Fluids and feeding |  |  |  |  |  |
| Normal diet |  |  |  |  |  |
| Soft diet |  |  |  |  |  |
| Nasogastric tube |  |  |  |  |  |
| Percutaneous feeding tube |  |  |  |  |  |
| Intravenous/subcutaneous fluids |  |  |  |  |  |
| No feeding/fluids |  |  |  |  |  |
| Qualifying event (%) † |  |  |  |  |  |
| Ischaemic stroke |  |  |  |  |  |
| Haemorrhagic stroke |  |  |  |  |  |
| Stroke type unknown |  |  |  |  |  |
| Non-stroke |  |  |  |  |  |
| Side of lesion, right (%) |  |  |  |  |  |
| SSS (/58) † |  |  |  |  |  |
| NIHSS (/42) ^19^ |  |  |  |  |  |
| GCS <15 (%) |  |  |  |  |  |
| Clinical syndrome ^24^ |  |  |  |  |  |
| TACS † |  |  |  |  |  |
| PACS |  |  |  |  |  |
| LACS |  |  |  |  |  |
| POCS |  |  |  |  |  |
| IS aetiology |  |  |  |  |  |
| Cardioembolic |  |  |  |  |  |
| Large vessel |  |  |  |  |  |
| Small vessel disease |  |  |  |  |  |
| Other |  |  |  |  |  |
| Haemodynamics |  |  |  |  |  |
| BP, Systolic (mmHg) † |  |  |  |  |  |
| BP, Diastolic (mmHg) |  |  |  |  |  |
| Heart rate (bpm) |  |  |  |  |  |
| OTR (hours) † |  |  |  |  |  |
| Thrombolysis (%)† |  |  |  |  |  |
| Continue/Stop Randomisation |  |  |  |  |  |
| Continue |  |  |  |  |  |
| Stop |  |  |  |  |  |
| Not relevant |  |  |  |  |  |
| Baseline scan (%) |  |  |  |  |  |
| Visible infarction |  |  |  |  |  |
| Visible haemorrhage |  |  |  |  |  |
| No lesion seen |  |  |  |  |  |
| Non-stroke lesion |  |  |  |  |  |

† Variable used in statistical adjustment

BP: blood pressure; bpm: beats per minute; GCS: Glasgow Coma Scale; IHD: ischaemic heart disease; IS: ischaemic stroke; LACS: lacunar syndrome; mRS: modified Rankin Scale; NIHSS: National Institutes of Health Stroke Scale; OTR: time from onset to randomisation; PACS: partial anterior circulation syndrome; PAD: peripheral artery disease; POCS: posterior circulation syndrome; SSS: Scandinavian Stroke Scale; TACS: total anterior circulation syndrome; TIA: transient ischaemic attack.

**Table 2**. Primary and secondary outcomes, and safety measures, at days 7 and 90 in patients randomised within 12 hours of stroke onset. Data are number (%), median [interquartile range] or mean (standard deviation). Comparisons between continue vs stop prior antihypertensives use binary logistic regression, ordinal logistic regression or multiple regression; results are odds ratio or mean difference, with 95% confidence intervals and significance.

|  | All | Continue | Stop | OR/MD (95%CI) | 2p |
| --- | --- | --- | --- | --- | --- |
| *Modified Rankin Scale* |  |  |  |  |  |
| Median (/6), primary outcome |  |  |  |  |  |
| mRS>2, adjusted |  |  |  |  |  |
| *Day 7* |  |  |  |  |  |
| SICH (%) |  |  |  |  |  |
| Recurrent stroke (%) |  |  |  |  |  |
| Deterioration (%) ^14^ |  |  |  |  |  |
| SSS (/58) |  |  |  |  |  |
| NIHSS (/42) ^19^ |  |  |  |  |  |
| *Hospital and discharge* |  |  |  |  |  |
| Length of stay (days) |  |  |  |  |  |
| Death or institution (%) |  |  |  |  |  |
| Speech therapy involvement (%) |  |  |  |  |  |
| *Day 90* |  |  |  |  |  |
| Barthel Index (/100) |  |  |  |  |  |
| EQ-5D/HUS (/1) |  |  |  |  |  |
| EQ-VAS (/100) (N=256) |  |  |  |  |  |
| MMSE (N=*) |  |  |  |  |  |
| TICS-M (N=*) |  |  |  |  |  |
| Verbal Fluency (/∞) (N=*) |  |  |  |  |  |
| ZDS (/100) (N=*) |  |  |  |  |  |
| Death or institution (%) |  |  |  |  |  |
| *Safety* |  |  |  |  |  |
| Patients with SAE (%) |  |  |  |  |  |
| Day 7 |  |  |  |  |  |
| Day 90 |  |  |  |  |  |
| Died (%) |  |  |  |  |  |
| By day 7 |  |  |  |  |  |
| In hospital |  |  |  |  |  |
| By day 90 |  |  |  |  |  |
| Day 7 (%) |  |  |  |  |  |
| Headache |  |  |  |  |  |
| Hypotension |  |  |  |  |  |
| Hypertension |  |  |  |  |  |
| Recurrence |  |  |  |  |  |

EQ-5D: *; EQ-VAS: *; mRS: modified Rankin Scale; NIHSS: National Institutes of Health Stroke Scale; SICH: symptomatic intracranial haemorrhage; SSS: Scandinavian Stroke Scale; ZDS: *

**Figure 1**. Comparison in distribution of seven-level modified Rankin Scale between continue versus stop prior antihypertensives at day 90: adjusted common odds ratio * (95% confidence interval *-*, p=*); unadjusted common odds ratio * (95% confidence interval *, *, p=*).

**Figure 2**. Subgroup analysis of effects on functional outcome at 90 days for continue versus stop prior antihypertensives for patients enrolled within 12 hours of stroke onset. Two-sided p values are for the adjusted interaction between subgroup and allocated treatment. OCSP: Oxfordshire Community Stroke Project.^24^

**Figure 3**. Survival curves over the 90 days of follow-up: continue versus stop prior antihypertensives: hazard ratio * (95% confidence interval *-*; p=*).

**Figure *.** Systolic and diastolic blood pressure over 7 days: continue versus stop pre-stroke antihypertensive drugs patients enrolled within 12 hours of stroke onset.

**Supplementary Table ***.

|  | Continue |  | 2p | Stop |  | 2p |
| --- | --- | --- | --- | --- | --- | --- |
| Feeding ^46^ | Oral | Non-oral |  | Oral | Non-oral |  |
| Patients |  |  |  |  |  |  |
| Pneumonia |  |  |  |  |  |  |
| Physiotherapy |  |  |  |  |  |  |
| Occupational therapy |  |  |  |  |  |  |
| Speech therapy |  |  |  |  |  |  |
|  |  |  |  |  |  |  |

**Supplementary Table *.** Number of patients with serious adverse events during follow-up to day 90: continue versus stop pre-stroke antihypertensive drugs in patients enrolled within 12 hours of stroke onset.

| Cause | Any |  | Fatal |  |
| --- | --- | --- | --- | --- |
|  | Continue | Stop | Continue | Stop |
| Complication of initial stroke |  |  |  |  |
| Extension of initial stroke |  |  |  |  |
| Symptomatic intracranial haemorrhage |  |  |  |  |
| Recurrent stroke |  |  |  |  |
| Myocardial infarction |  |  |  |  |
| Sudden cardiac death | **-** | **-** |  |  |
| Atrial fibrillation |  |  |  |  |
| Carotid endarterectomy |  |  |  |  |
| Other cardiovascular event |  |  |  |  |
| Pulmonary embolism |  |  |  |  |
| Pneumonia |  |  |  |  |
| Other event |  |  |  |  |
| Total |  |  |  |  |

Data are number of patients (%). Comparison by Chi-square test: † P<0.05; all other comparisons are non-significant. Definitions for some events are given above, and in the ENOS Statistical Analysis Plan.

**Supplementary Figure ***. Shift figure of SAES (fatal, non-fatal SAE, no SAE) by continuous vs stop prior antihypertensive agents. Comparison by ordinal logistic regression: Odds ratio * (95% confidence intervals *-*; p=*).
